# Difference between Impacts of COVID-19 on Women and Men’s Psychological, Social, Vulnerable Work Situations, and Economic Well-Being

**DOI:** 10.3390/ijerph19148849

**Published:** 2022-07-21

**Authors:** Enrique Iglesias Martínez, Jorge Roces García, Estibaliz Jiménez Arberas, José Antonio Llosa

**Affiliations:** 1 Social Education, Faculty Padre Ossó, University of Oviedo, 33008 Oviedo, Spain; iglesiasenrique@uniovi.es (E.I.M.); llosajose@uniovi.es (J.A.L.); 2 Polytechnic School of Engineering of Gijón, University of Oviedo, 33204 Gijón, Spain; 3 Occupational Therapy, Faculty Padre Ossó, University of Oviedo, 33008 Oviedo, Spain; jimenezestibaliz@uniovi.es

**Keywords:** social determinants, mental health, anxiety, women, sanitary crisis, SARS-CoV-2

## Abstract

The SARS-CoV-2 virus changed social reality worldwide, affecting people’s health and work life, particularly their anxiety levels. The purpose of this study is to verify the situation of women in terms of anxiety and social determinants in Spain during the pandemic. The sample consisted of 4686 people (3500 women and 1186 men). The tools used were the State-Trait Anxiety Inventory (STAI) and an ad hoc questionnaire to assess the work and mental situation of the participants. The results indicate a higher rate of anxiety among women than among men and reveal a relationship between higher levels of anxiety and more vulnerable work situations in terms of higher unemployment rates, contract changes, etc. Furthermore, there was a higher percentage of women than men in the sectors where the health crisis has had a greater impact and presence, with repercussions on the physical, mental, and social health of the entire population and especially on women. It is necessary to take into account the social determinants of health, not only at the structural level, in terms of the socio-economic and political contexts, to avoid and limit the axes of inequality such as gender.

## 1. Introduction

The health situation caused by the SARS-CoV-2 virus in 2020 completely changed social reality. On 11 March 2020, the World Health Organization called for exceptional measures and declared the situation an international pandemic [1].

Due to the health situation and cases of infection, the world population has been forced, based on the criteria established by health authorities, to reduce travel, take precautions to reduce the transmission of the virus, and stay at home as basic means to limit exposure to the virus [2]. In Spain, on 14 March 2020, a state of alarm was declared throughout the national territory to protect the health and safety of citizens, contain the progression of the disease, and strengthen the public health system [3].

The direct and indirect psychological and social consequences of the pandemic were widespread and affected mental health [4] because these measures impacted the habits, routines, and roles performed by individuals in day-to-day life [5,6]. Analyzing these adaptations and knowing their consequences is of great interest because “staying homebound”, although considered a safety measure in terms of preventing possible infection, can possibly have a considerable psychological impact [7]. Indeed, it has been observed that during the first days of the pandemic, psychological problems, including anxiety and stress, increased directly as a result of the pandemic [8,9] and indirectly as a result of the costs associated with concerns regarding the economic and social situation [10], a very relevant fact in itself that can also have repercussions on various aspects of health [11].

The term anxiety refers to an emotional reaction that arises when people face unfamiliar situations [12]. However, Spielberger [13,14] considers that to conceptualize anxiety correctly, it is necessary to differentiate between anxiety as a personality trait and anxiety as an emotional state. According to Spielberger, *state anxiety* (S/A) is an immediate and transitory emotional state that can be modified across time and is perceived as a combination of tension, apprehension, nervousness, and worry, in combination with physiological changes. *Trait anxiety* (T/A) refers to a disposition, tendency, or trait, explained as a predisposed behavior of an individual to perceive a large number of situations as threatening; T/A has characteristics of chronicity.

As concluded in numerous studies, women are more likely to develop mental illnesses such as anxiety [15,16,17,18], and women are more affected during pandemics [19]. Additionally, the World Health Organization (WHO) prioritizes mental health among its objectives and foresees a mental health crisis resulting directly from the COVID-19 pandemic [1]. Therefore, society is on the precipice of an increased risk of mental health issues; as such, analyses of the mental health and socio-economic situation of women during periods of confinement are of great interest. Because the mental health needs of patients with confirmed COVID-19, patients with suspected infection, and people in quarantine seem to have been poorly managed [20], with these individuals not receiving necessary care, it is essential to understand the psychological consequences of self-isolation during the pandemic [8].

Therefore, the objective of this study was to analyze the anxiety level, employment status, and socio-economic status of women during the state of alarm caused by the COVID-19 pandemic in Spain.

## 2. Method

### 2.1. Participants

Quota and convenience sampling were conducted. The target sample of this study was comprised of men and women in confinement at the time of answering the questionnaire. A total of 4686 individuals were included; 74.7% were women (n = 3500), and 26.3% were men (n = 1186), aged between 18 and 72 years (M = 37.90 years, *SD* = 12.46 years).

### 2.2. Procedure

A cross-sectional, ex post facto survey design was adopted to evaluate the immediate psychological response during confinement throughout the Spanish territory. The survey was disseminated electronically through different universities. Data collection began on 22 March 2020, 8 days after the start of home confinement in Spain, and responses were accepted until 24 May 2020, the date that home confinement was relaxed. The purpose was to collect data for the period during which the confinement of the population was stricter. All participants took part voluntarily and gave their consent after having been informed about the objectives and methodology of the research project. Their anonymity was respected, and data confidentiality was guaranteed. Consequently, they were not asked for identification variables. This research follows the requirements and protocols of the Ethical Committee of the Psychology Department of the university where it was carried out and the recommendations of the Declaration of Helsinki.

### 2.3. Instruments

Sociodemographic information was collected using an ad hoc form. The following data were collected: gender, age, and employment status (employee, student, retired, civil servant, unemployed, self-employed, family tasks, and other). Additionally, the following questions were asked: *Do you have a job? What is your monthly salary? Do you have to leave home to go to work? Have you undergone a contractual change? (none, record of temporary employment regulation (ERTE, acronym in Spanish), forced vacation, fired, and salary increase)? Have you had mental health problems?*

To collect data on anxiety, the Spanish adaptation of the State-Trait Anxiety Inventory (STAI) was used [21]. The instrument consists of 2 parts, with 20 questions each. The first part, State Anxiety (S/A), evaluates a transitory emotional state characterized by subjective feelings, consciously perceived, of attention and apprehension generated through hyperactivity of the autonomic nervous system. The second part, Trait Anxiety (T/A), evaluates an anxious propensity that is relatively chronic and causes individuals to perceive situations as threatening. The application time is approximately 20 min. The psychometric guarantees of the tool have good internal consistency, with a Cronbach’s alpha for this study of 0.93 for the state scale and 0.87 for the trait scale. The internal consistency of the STAI scales is optimal.

### 2.4. Data Analysis

First, a descriptive analysis of the variables and groups analyzed was performed. Secondly, the variables determined as qualitative were analyzed with Pearson’s chi-square and Fisher’s Test. On the other hand, comparisons of means were developed with t-Student (NC 95%) and ANOVA (NC 95%) in the comparison of two or three or more groups, respectively. In the comparisons with three or more groups, Tukey’s test (NC 95%) was also performed to determine in which groups the significant differences were located. Finally, a multivariate linear model (95% NC) was designed to establish the variables associated with anxiety detected as a trait or state and to study whether the variables that determine the presence of each construct differ. All analyses were carried out with *The R Project for Statistical Computing* (R Development Core Team. It is seated Vienna, Austria, and is active worldwide) version 3.6.0.

## 3. Results

Descriptive Statistics and Bivariate Correlations

In the 4,686 responses analyzed, higher frequencies of trait anxiety (74.88%) than trait anxiety (53.61%) were observed. The exploratory analysis shows, in any case, a high incidence of both anxiogenic phenomena.

There was an association between *telework* and *gender* (Pearson’s chi-square test, *p*-value = 0.008); a higher percentage of women than men had in-person jobs (Table 1).

There was an association between *profession* and *gender* (Fisher’s test, *p*-value < 0.001); a higher percentage of women than men worked in health, services, and education (Table 2).

There was an association between *employment status* and *gender* (Pearson’s chi-square test, *p*-value < 0.001); higher percentages of women than men performed family tasks and were unemployed (Table 3).

There was an association between *monthly salary* and *gender* (Pearson’s chi-square test, *p*-value < 0.001). Women had less income than men. There was a relationship between being a woman and not having an income or earning less than EUR 1200, and there was a positive relationship between being a man and earning more than EUR 1200. There was a negative relationship between being a woman and earning more than EUR 1200 and more than EUR 1660 (Table 4).

There was an association between *contractual changes* and *gender* (Pearson’s chi-square test, *p*-value = 0.057). A higher percentage of women than men were fired, resulting in a positive correlation between the variables *woman* and *fired;* in contrast, the variables *man* and *fired* were negatively correlated (Table 5).

Also, there was an association between *Have you had mental health problems?* and *gender* (Pearson’s chi-square test, *p*-value < 0.001); women reported more mental health problems than did men. Likewise, there was an association between *anxiety treatment* and *gender* (Pearson’s chi-square test, *p*-value < 0.001) (Table 6).

Finally, there was an association between *gender* and *state anxiety* (Pearson’s chi-square test, *p*-value < 0.001); women had higher levels of anxiety than did men. (Table 7).

The following summary values (all quantitative) for the measured variables are presented. The number of available data, mean, standard deviation, 0 percentile or the minimum value, 25th percentile or 1st quartile, 50th percentile or median, 75th percentile or 3rd quartile, and 100th percentile or the maximum value. The difference between STAI state (S/T) and STAI trait is denoted by *dif.* (Table 8).

To determine whether confinement had influenced anxiety, the difference between S/A and T/A was analyzed. Among the 4686 respondents, the mean was 6.82 units, with a standard deviation of 10.23 units; the median was 7 units. Given that there was a sufficient sample size (n = 4686), the results from the test performed indicated that there were differences between the variables (Student’s *t*-test, *p*-value < 0.001) (Table 9). The results of these two analyses allow an examination of the means. It shows that state anxiety scores are higher than trait anxiety scores, which denotes a consequence of the COVID-19 situation that these individuals were going through.

## 4. Discussion

The global pandemic caused by COVID-19 has had and will have health and economic repercussions, and importantly, the psychological impact of the health crisis will endure [22]. The most reported psychological consequences are depression, anxiety, and related symptoms [23,24]. However, although anxiety levels seem to have increased for the entire population, the results of this study indicate a higher rate of anxiety among women during the pandemic, a finding that is consistent with that reported in Books et al. (2020) [7] and Iglesias-Martínez et al. [25], as well as in studies that reported that women presented levels of anxiety three times higher than those presented by men during the state of alarm [26].

The restrictions implemented because of the pandemic have had a substantial impact on employment and have caused greater inequality in employment [27]. Among the consequences, contractual changes and an increase in the labor gap between men and women were observed in this study [28,29]. Previous studies, such as that by Gálvez Muñoz and Rodrígez Modroño (2011) [30], concluded that women are the most disadvantaged with regard to labor during economic crises, and these authors reported historical guidelines to guarantee equality. In addition, the results obtained in this study indicate that the general employment situation of women is more precarious because they are employed in sectors where the crisis has had a greater presence and a greater impact, such as health, education, and the service sector, due to the decrease in demand and to the implementation of restrictions [31,32,33,34].

Health professionals, particularly those who are female, seem to be at a greater predisposition to suffer from certain mental illnesses during stressful situations [35,36], as supported by the findings in this study and those reported by previous studies such as [32,37].

The cause of this higher rate and a higher level of anxiety among females can be explained by several reasons, among which work environment and the need to support the family stand out [38], factors that have been even more pronounced in the context of confinement during the state of alarm. The majority of women in the present study worked in person despite the risks and stressors that this entailed, e.g., fear of infection (themselves and others) and fear stemming from myths and disinformation on social networks and through other media [39]. However, teleworking, although it has advantages, can perpetuate the caregiver role played by women [40,41]. Currently, compared with men [42], women dedicate twice as much time to the home and family, a situation that increases levels of anxiety and worsened in this pandemic [25,43].

The data obtained in the study indicate that women have suffered from more vulnerable work situations and have a higher rate of unemployment in Spain, a finding consistent with that reported in other studies, such as [27]. A greater number of women than men did not have an income during confinement and, therefore, experienced more consequences, a finding that is consistent with those reported by previous studies showing that women, compared with men, are more negatively affected by pandemics [19].

This work reveals that economic inequality is a synergistic factor, a finding that requires the development of specific conceptual frameworks for the implementation of prevention and control programs to address comorbidities [44]. Therefore, public health policies must be implemented to ensure that the COVID-19 pandemic does not cause long-term increases in social inequality among future generations [45] and especially in women, as shown in this study, and to ensure that mental health is taken into account.

Among the limitations of this study, the sampling method and the study design do not allow the extrapolation of results to other populations but did allow an estimate of the mental health of women during the state of alarm. In future lines of research, there is a need to reproduce and increase the number of studies that highlight the economic and social vulnerability of women and that can be used for the development of measures that address, among other issues, employment opportunities, family support, and mental health for females.

## 5. Conclusions

The health situation caused by COVID-19 has had and will have repercussions on the physical, mental and social health of the entire population. It is necessary to take into account the social determinants of health, not only at the structural level, in terms of the socio-economic and political contexts, to avoid and limit the axes of inequality such as gender, social class, or even age, but also through macroeconomic policies that minimize the sequelae and consequences on employment and work conditions, income and economic situations, and domestic and care work because all these determinants have an impact on health inequalities. The results obtained in the present study can be extrapolated to the Spanish population; they reflect both a greater salary gap during confinement than during previous periods in our country and a worsening of the mental health of females. These differences trigger a negative impact on quality of life, and the long-term repercussions of that impact are unknown.

## Figures and Tables

**Table 1:** Relationship between telework and gender.

	Men			Women			Chi-Square
	n	%Col	%Row	n	%Col	%Row	*p*
In-person	521	64.56	30.13	1208	59.16	69.87	0.008 *
Telework	286	35.44	25.54	834	40.84	74.46	

Note. Chi-square test (<0.05; * <0.01).

**Table 2:** Relationship between profession and gender.

	Men			Women		
	n	%Col	%Row	n	%Col	%Row
Security forces	11	1.79	78.57	3	0.13	21.43
Banking	9	1.46	23.08	30	1.34	76.92
Construction	16	2.60	34.04	31	1.39	65.96
Education	151	24.55	21.42	554	24.83	78.58
Industrial	76	12.36	44.44	95	4.26	55.56
Other	139	22.60	19.97	557	24.97	80.03
Health	71	11.54	13.76	445	19.95	86.24
Services	142	23.09	21.58	516	23.13	78.42

Note. Fisher’s test (*p*-value < 0.001).

**Table 3:** Relationship between employment status and gender.

	Men			Women		
	n	%Col	%Row	n	%Col	%Row
Employee	495	41.70	25.00	1485	42.44	75.00
Self-employed	122	10.28	35.36	223	6.37	64.64
Student	208	17.52	27.30	554	15.83	72.70
Official	159	13.40	24.50	490	14.00	75.50
Retired	61	5.14	44.85	75	2.14	55.15
Other	53	4.47	24.77	161	4.60	75.23
Unemployed	84	7.08	17.43	398	11.37	82.57
Family tasks	5	0.42	4.24	113	3.23	95.76

Note. Pearson’s chi-square test (*p*-value < 0.001).

**Table 4:** Monthly salary and gender.

	Men			Women		
	n	%Col	%Row	n	%Col	%Row
No income	218	18.37	22.54	749	21.41	77.46
Less than EUR 500	78	6.57	20.86	296	8.46	79.14
Between EUR 500 and EUR 800	62	5.22	13.87	385	11.00	86.13
Between EUR 800 and EUR 1200	211	17.78	19.34	880	25.15	80.66
Between EUR 1200 and EUR 1600	258	21.74	28.99	632	18.06	71.01
More than EUR 1600	360	30.33	39.26	557	15.92	60.74

Note. Pearson’s chi-square test (*p*-value < 0.001).

**Table 5:** Relationship between contractual changes and gender.

	Men			Women		
	n	%Col	%Row	n	Col	%Row
None	916	77.17	25.37	2694	76.99	74.63
Fired	31	2.61	17.32	148	4.23	82.68
ERTE *	155	13.06	25.41	455	13.00	74.59
Forced vacation	79	6.66	30.04	184	5.26	69.96
Salary increase	6	0.51	25.00	18	0.51	75.00

* Temporary employment regulation. Note. Pearson’s chi-square test (*p*-value = 0.057).

**Table 6:** Relationship between mental health, anxiety treatment, and gender.

		Men			Women		
		n	%Col	%Row	n	%Col	%Row
Mental health problems	No	1.005	84.67	28.10	2572	73.51	71.90
	Yes	182	15.33	16.41	927	26.49	83.59
Anxiety treatment	No	907	76.73	30.41	2076	59.42	69.59
	Yes	275	23.27	16.24	1418	40.58	83.76

Note. Pearson’s chi-square test (*p*-value < 0.001).

**Table 7:** Relationship between state anxiety, gender, and mental health.

		Absence State Anxiety			Presence State Anxiety		
		n	%Col	%Row	n	%Col	%Row
Relationship mental health and state anxiety	No	901	76.62	30.20	2.082	59.49	69.80
	Yes	275	23.38	16.24	1.418	40.51	83.76
Relationship gender and state anxiety	Men	386	32.80	32.52	801	22.83	67.48
	Women	791	67.20	22.61	2708	77.17	77.39

Note. Pearson’s chi-square test (*p*-value < 0.001).

**Table 8:** Difference between STAI trait and STAI state.

				Percentile (%)				
	n	Media	D.t	0	25	50	75	100
STAI State	4686	29.63	11.59	0.00	21.00	30.00	38.00	60.00
STAI Trait	4686	22.81	9.97	0.00	16.00	22.00	29.00	56.00
Difference	4686	6.82	10.23	−37.00	0.00	7.00	13.00	53.00

**Table 9:** Difference between state anxiety and trait anxiety.

	n	Mean	Median	D.t	P25	P75
S/A	4686	29.63	30.00	11.58	21.00	38.00
T/A	4686	22.81	22.00	9.97	16.00	29.00
Difference	4686	6.81	7.00	10.22	0.00	13.00

Note. Student’s *t*-test (*p*-value < 0.001).

## Data Availability

The data presented in this study are available on request from the corresponding author.
